# Safety of Janus kinase inhibitors in rheumatoid arthritis: a disproportionality analysis using FAERS database from 2012 to 2022

**DOI:** 10.1007/s10067-025-07360-9

**Published:** 2025-02-13

**Authors:** Bahar Mikaeili, Zuhair A. Alqahtani, Ana L. Hincapie, Jeff Jianfei Guo

**Affiliations:** 1 Division of Pharmacy Practice & Administrative Sciences, James L. Winkle College of Pharmacy, University of Cincinnati, Cincinnati, OH USA; 2 College of Pharmacy, Health Economics and Outcome Research, King Saud University, Riyadh, Saudi Arabia

**Keywords:** Adverse events, Disproportionality analysis, FAERS, Janus kinase inhibitors, Rheumatoid arthritis, Safety

## Abstract

**Introduction/objective:**

Janus kinase (JAK) inhibitors have expanded treatment options for rheumatoid arthritis (RA), particularly for patients unresponsive to traditional disease-modifying antirheumatic drugs (DMARDs). However, safety concerns necessitate a thorough post-market evaluation. This study is aimed at comparing the safety profiles of tofacitinib, baricitinib, and upadacitinib using adverse event (AE) reports from the FDA Adverse Event Reporting System (FAERS).

**Methods:**

A retrospective disproportionality analysis was performed using the FAERS data from 2012 to 2022. The AE reports were categorized into cardiovascular, cancer, respiratory, gastrointestinal, musculoskeletal, and arthralgia-related events. Proportional reporting ratio (PRR) and reporting odds ratios (RORs) with 95% confidence intervals (CIs) were calculated to identify significant safety signals.

**Results:**

Of 273,657 AE reports, tofacitinib had the most (227,144), with increased musculoskeletal-related events (ROR = 1.53, 95% CI 1.49–1.57) and a reduced cancer risk (ROR = 0.44, 95% CI 0.41–0.47). Baricitinib (9305 reports) showed the highest risk of cardiovascular events (ROR = 1.63, 95% CI 1.50–1.78) and cancer (ROR = 2.17, 95% CI 1.83–2.58). Upadacitinib (37,208 reports) had elevated risks for respiratory events (ROR = 2.04, 95% CI 1.88–2.21) and cancer (ROR = 2.24, 95% CI 2.05–2.43).

**Conclusion:**

The distinct safety profiles of these JAK inhibitors suggest that baricitinib poses higher cardiovascular and cancer risks, whereas upadacitinib increases the risk of respiratory and gastrointestinal events. Tofacitinib may be safer for patients with a history of cancer but requires monitoring for musculoskeletal AEs. Personalized risk assessments are critical for safe use of JAK inhibitors.

**Table:** 

**Key Points** • *This study provides a comprehensive post-market safety assessment of three JAK inhibitors—tofacitinib, baricitinib, and upadacitinib—using the FAERS data from 2012 to 2022.* • *Distinct safety profiles were identified, with baricitinib showing a higher risk of cardiovascular events and cancer, while upadacitinib posed an increased risk of respiratory and gastrointestinal events.* • *Tofacitinib demonstrated a lower cancer risk than other JAK inhibitors but was associated with more musculoskeletal-related adverse events.* • *These findings emphasize the importance of personalized risk assessment and vigilant monitoring when prescribing JAK inhibitors for rheumatoid arthritis.*

## Introduction

Rheumatoid arthritis (RA) is a chronic autoimmune disease characterized by persistent inflammation. It affects between 0.5 and 1% of the adult population and is observed two to three times more frequently in women than in men [1, 2]. The etiology of RA involves a complex interplay of genetic, environmental, and immunological factors, highlighting the need for multifaceted treatment approaches [3]. Significant advancements have been made in treating RA, particularly with the development of disease-modifying antirheumatic drugs (DMARDs), which play a crucial role in managing the disease [4, 5]. Traditional therapies, such as methotrexate, aim to slow disease progression and mitigate symptoms [6]. However, the varied response rates and potential adverse events associated with DMARDs have led to the development and adoption of novel treatment modalities [7].

While traditional DMARDs like methotrexate have been foundational in RA management, recent advancements have introduced Janus kinase (JAK) inhibitors as a groundbreaking alternative. Tofacitinib (Xeljanz) received FDA approval in 2012, followed by baricitinib (Olumiant) in 2018, and upadacitinib (Rinvoq) in 2019 [8–10]. These drugs have been lauded for their efficacy in controlling RA symptoms, offering new hope to patients who have not responded adequately to traditional or biologic DMARDs. However, emerging safety concerns have tempered the excitement surrounding these new treatments. The FDA has highlighted significant adverse events associated with JAK inhibitors, including major adverse cardiovascular events (MACE), thrombosis, malignancies, and serious infections [9–12]. A notable safety trial for tofacitinib concluded increased risks of heart-related events and cancer, prompting a re-evaluation of its use, particularly among patients who have not responded to TNF inhibitors, which fall under the category of biologic DMARDs [13, 14]. Preliminary findings also suggest an elevated risk of MACE and thrombosis with baricitinib, with these concerns extended to upadacitinib due to their similar mechanisms of action [15–18]. These developments underscore the critical need for comprehensive safety assessment of these medications.

Despite these advances and concerns, a gap remains in the literature regarding a comprehensive examination of the safety profiles of tofacitinib, baricitinib, and upadacitinib from 2012 to 2022. Thus, this study is aimed at evaluating adverse event rates to provide insights into the comparative safety of tofacitinib, baricitinib, and upadacitinib. In doing so, it seeks to inform clinical decision-making and enhance patient care in the management of rheumatoid arthritis. The objective of this study was to analyze and quantify adverse events (AEs) reports associated with the use of JAK inhibitors (tofacitinib, baricitinib, and upadacitinib) using data from the FDA’s Adverse Event Reporting System (FAERS).

Additionally, the lack of detailed patient-level data in FAERS, such as duration of drug use and comorbidities, limits causal interpretations of the adverse events reported.

## Methods

### Study design and data source

A retrospective disproportionality analysis was carried out on all reported AEs associated with JAK inhibitors utilizing FAERS database between January 2012 and December 2022. The FDA receives voluntary reports on AEs, medication errors, and product quality concerns from consumers, manufacturers, and healthcare professionals during clinical trials and after approved drugs are marketed. These reports are archived in the publicly accessible FAERS Drug Safety Monitoring Database [19]. The FDA defines an AE as any side effect related to the use of a drug in humans, whether or not it is thought to be drug-related. This includes any AE that occurs when using a drug in professional practice, unintentional or intentional drug overdose, drug abuse, withdrawal symptoms, adverse events from drug abuse, and failure to achieve anticipated pharmacological action. Adverse event cases are defined as event reports, including de-identified patient demographics (age and sex), suspected medication, treatment indication, type of event, results, and, if relevant, manufacturer information. This study did not involve human participants, eliminating the need for both individual consent and approval from an institutional review board. It is important to note that FAERS does not capture the duration of drug use, the temporal relationship between drug administration and AE occurrence, or concomitant medications, which are potential confounders in assessing drug safety.

### Measures and operational definitions

Janus kinase (JAK) inhibitors listed in Table 1 were queried using their brand and generic names. According to the FDA’s recommendation, duplicate records based on the primary AE case report identifying number (“primary_id”) and any follow-up reports were eliminated [20]. The variable “preferred_term,” or “PT,” was used to retrieve AEs from the database. The most frequently reported AEs were examined and categorized based on disease categories, including musculoskeletal, respiratory, arthralgia, cardiovascular, gastrointestinal, and cancer. Furthermore, clinical outcomes defined by FAERS (initial or prolonged hospitalization, life-threatening, disability, congenital anomaly, requiring immediate intervention, and death) were retrieved and examined.

**Table 1:** JAK inhibitors included in the study and their FDA approval dates

Generic (brand)	Initial FDA approval date
Tofacitinib (Xeljanz®)	November 06, 2012
Baricitinib (Olumiant®)	June 01, 2018
Upadacitinib (Rinvoq®)	August 16, 2019

### Data analysis

Descriptive analysis was used to express patients’ demographics (age and sex), total number of AE reports, AE counts, and clinical outcomes for each drug. Moreover, a disproportionality analysis based on the proportional reporting ratio (PRR) and reporting odds ratio (ROR) was carried out to identify safety signals for a specific JAK inhibitor. The PRR and ROR, along with their 95% confidence intervals (CIs), were computed using two-by-two contingency tables for the number of the most frequently reported AEs and clinically significant outcomes [21]. The proportion of AE of interest for one JAK inhibitor compared to the other two was used to establish the relationship between the given drug and the targeted AE. A signal was identified if the number of targeted events was at least 3. For ROR, a positive signal was identified if the lower limit of the 95% CI was $>$ 1. For PRR, a positive signal was identified if the PRR was ≥ 2 [22]. The SAS software package for Windows (SAS Institute Inc., Version 9.4) and Microsoft Excel were used for all the analyses.

## Results

Table 2 summarizes the demographic characteristics of case patients of each JAK inhibitor. Most of the patients were female across all three JAK inhibitor reports. The mean ages were similar, with patients generally in their mid- to late 50 s.

**Table 2:** Case patients’ demographics in FAERS reports for tofacitinib, baricitinib, and upadacitinib adverse events *

Demographics	Drug		
	Tofacitinib	Baricitinib	Upadacitinib
**Mean age, y (SD)**	57.5 (6.15)	60.14 (2.96)	58.5 (1.31)
Females* (%)	14,252 (75.68)	697 (62.34)	4683 (72.46)
**Males (%)**	3398 (18.04)	346 (30.95)	1434 (22.19)
**Unknown (%)**	1182 (6.28)	75 (6.71)	346 (5.35)

^*^ # of reports

### Frequency of adverse events reports

**Fig. 1:**
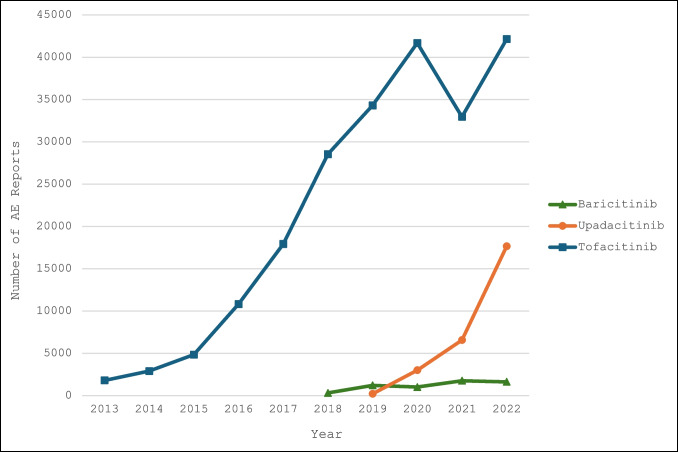
Total number of adverse event reports of tofacitinib, baricitinib, and upadacitinib by year

Figure 1 illustrates the annual variability in the total number of AE reports for each JAK inhibitor from 2013 to 2022. Tofacitinib consistently had the highest number of reports, peaking at 42,140 in 2022. Upadacitinib showed a steady increase in AE reports, reaching a peak of 17,648 in 2022. Baricitinib exhibited a gradual increase, peaking at 1751 in 2021. The most frequently reported AEs across all JAK inhibitors included cardiovascular, cancer, arthralgia, respiratory, musculoskeletal, and gastrointestinal diseases.

### Clinical adverse events and outcomes

**Fig. 2:**
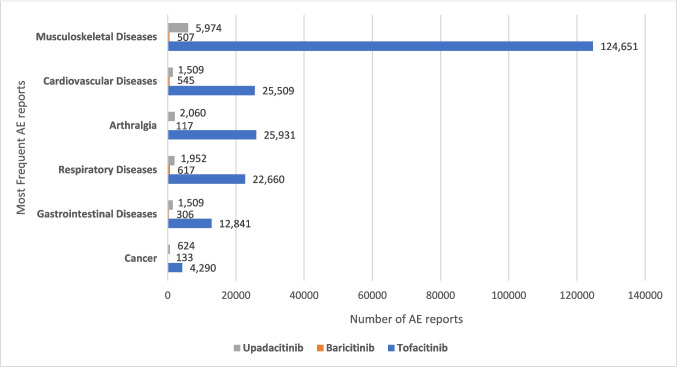
Most frequent adverse event reports for tofacitinib, baricitinib, and upadacitinib from 2013 to 2022

Figure 2 illustrates the most common adverse events for each JAK inhibitor. The distribution of these AEs demonstrated year-to-year variability. Across the study period, each drug had a specific profile, with tofacitinib and upadacitinib showing a high frequency of musculoskeletal-related events (124,651 and 5974 reports, respectively), while baricitinib had a higher frequency of cardiovascular-related events (545 reports).

As illustrated in Table 3, adverse event outcomes reported varied between tofacitinib, baricitinib, and upadacitinib, which included hospitalization, disability, life-threatening events, death, congenital anomaly, and other serious outcomes. Adverse events that led to hospitalizations were higher across all JAK inhibitors (27,399 reports for tofacitinib, 2257 for baricitinib, and 6275 for upadacitinib). In addition, disability outcomes and life-threatening events were higher among all three JAK inhibitors, with disability reports of 7282 tofacitinib, 127 baricitinib, and 298 upadacitinib, and life-threatening reports of 3137 tofacitinib, 414 baricitinib, and 199 upadacitinib.

**Table 3:** Most frequently reported adverse event outcomes for tofacitinib, baricitinib, and upadacitinib from 2013 to 2022

Drug/outcome	Congenital anomaly	Death	Disability	Hospitalization	Other serious	Life-threatening	Required intervention
Tofacitinib	269	5102	7282	27,399	90,608	3137	34
Baricitinib	5	602	127	2257	2387	414	94
Upadacitinib	5	887	298	6275	10,849	199	13

### Disproportional analyses

The disproportionality analyses presented in Table 4 revealed significant differences in the risk profiles of the three JAK inhibitors. Baricitinib had the highest reporting odds ratio for cardiovascular-related events (ROR = 1.6308, 95% CI 1.4962, 1.7776). Upadacitinib showed the highest reporting odds ratio of cancer (ROR = 2.235, 95% CI 2.0547, 2.4312) compared to baricitinib (ROR = 2.1704, 95% CI 1.8258, 2.5801) and tofacitinib (ROR = 0.4377, 95% CI: 0.405, 0.4729). Baricitinib also had the highest reporting odds ratio for respiratory-related events (ROR = 2.0392, 95% CI 1.8799, 2.212). Tofacitinib had the highest reporting odds ratio for musculoskeletal-related events (ROR = 1.5277, 95% CI 1.4889, 1.5675), while upadacitinib showed the highest reporting odds ratio for gastrointestinal-related reports (ROR = 1.8177, 95% CI 1.7226, 1.918). Upadacitinib also had a higher reporting odds ratio for arthralgia events (ROR = 1.254, 95% CI 1.1983, 1.3122) compared to tofacitinib and baricitinib.

**Table 4:** Disproportionality analyses for to facitinib, baricitinib, and upadacitinib

Adverse event	Tofacitinib		Baricitinib		Upadacitinib	
	ROR	PRR	ROR	PRR	ROR	PRR
**Cardiovascular diseases**	0.9625 (0.9197; 1.0072)	0.9632 (0.9213; 1.007)	1.6308 (1.4962; 1.7776)	1.6109 (1.4819; 1.7511)	0.9122 (0.8656; 0.9612)	0.9137 (0.8679; 0.962)
**Cancer**	0.4377 (0.405; 0.4729)	0.4395 (0.4069; 0.4747)	2.1704 (1.8258; 2.5801)	2.1614 (1.8206; 2.566)	2.235 (2.0547; 2.4312)	2.2258 (2.0474; 2.4196)
**Respiratory diseases**	0.6785 (0.651; 0.7071)	0.6841 (0.6571; 0.7122)	2.0392 (1.8799; 2.212)	2.002 (1.8509; 2.1654)	1.3308 (1.27; 1.3944)	1.323 (1.264; 1.3848)
**Arthralgia**	0.9111 (0.8719; 0.9521)	0.9129 (0.8745; 0.953)	0.3292 (0.2744; 0.395)	0.3338 (0.2785; 0.4)	1.254 (1.1983; 1.3122)	1.2477 (1.1936; 1.3042)
**Musculoskeletal diseases**	1.5277 (1.4889; 1.5675)	1.4769 (1.4418; 1.5127)	0.3274 (0.3011; 0.3559)	0.3497 (0.3226; 0.3791)	0.7335 (0.714; 0.7535)	0.7526 (0.7341; 0.7717)
**Gastrointestinal diseases**	0.527 (0.5019; 0.5534)	0.5317 (0.5068; 0.5579)	2.0478 (1.8434; 2.2748)	2.0257 (1.8275; 2.2453)	1.8177 (1.7226; 1.918)	1.8029 (1.7101; 1.9006)

All the *P*-values for all adverse events are < 0.001, except for cardiovascular events for tofacitinib

## Discussion

This study provides a comprehensive evaluation of adverse events associated with the JAK inhibitors—tofacitinib (tofacitinib), baricitinib (baricitinib), and upadacitinib (upadacitinib)—in the treatment of rheumatoid arthritis utilizing the publicly available data from the FAERS between 2012 and 2022. The findings revealed distinct safety profiles for these medications, highlighting the necessity for individualized risk assessment and careful patient selection in clinical practice. Our analysis identified significant differences in the frequency and types of AEs reported for each JAK inhibitor, which have important implications for their use in different clinical scenarios.

Tofacitinib, the first JAK inhibitor approved for RA, had the highest number of reported AEs, likely reflecting its longer time on the market and broader use compared to baricitinib and upadacitinib. The most frequently reported AEs for tofacitinib were musculoskeletal-related events (124,651 reports) and arthralgia (25,931 reports), with a notably high ROR for musculoskeletal events (ROR = 1.53; 95% CI 1.49–1.57). This suggests that patients receiving tofacitinib may have experienced more musculoskeletal-related AEs compared to those using other JAK inhibitors. Previous studies have reported similar findings, with musculoskeletal symptoms, such as arthralgia and myalgia, being common in patients treated with tofacitinib [23].

Interestingly, tofacitinib was associated with a lower risk of cancer (ROR = 0.44; 95% CI 0.41–0.47) than baricitinib and upadacitinib. This finding may make tofacitinib a preferable option for patients with a history of malignancy or for those at a higher risk for cancer. However, the high incidence of gastrointestinal events (12,841 reports) indicates that gastrointestinal monitoring is essential when prescribing tofacitinib. Studies have highlighted an increased risk of serious infections and gastrointestinal perforations with tofacitinib, therefore necessitating vigilance among patients with pre-existing gastrointestinal conditions [24].

Baricitinib demonstrated the highest reporting odds ratio for cardiovascular (ROR = 1.63; 95% CI 1.50–1.78) and cancer-related events (ROR = 2.17; 95% CI 1.83–2.58) among the three JAK inhibitors. This suggests that baricitinib may pose a higher risk of major adverse cardiovascular events (MACE) and malignancies than other JAK inhibitors. These observations are consistent with findings from previous clinical trials and observational studies. For instance, a study by Taylor et al. reported an increased incidence of cardiovascular events in patients treated with baricitinib (baricitinib) [25]. Moreover, the FDA has issued safety communications regarding the potential increased risk of MACE and malignancies associated with JAK inhibitors, particularly in patients with risk factors [26]. Therefore, patients with pre-existing cardiovascular conditions or a history of cancer may be at an increased risk when using baricitinib. Clinicians should exercise caution when considering baricitinib for patients with cardiovascular risk factors, such as hypertension, hyperlipidemia, or a history of cardiovascular disease. Alternative therapies with a lower cardiovascular risk profile may be preferable in these patients.

Upadacitinib, although newer to the market, showed a concerning number of respiratory (ROR = 2.04; 95% CI 1.88–2.21) and gastrointestinal-related events (ROR = 1.82; 95% CI 1.72–1.92). Additionally, upadacitinib had the highest ROR for cancer among the three drugs (ROR = 2.24; 95% CI 2.05–2.43). The increased incidence of respiratory events with upadacitinib suggests that patients with pre-existing respiratory conditions such as chronic obstructive pulmonary disease or asthma may require careful monitoring or may be better treated with alternative therapies. This finding aligns with reports from clinical trials, in which upadacitinib was associated with an increased risk of upper respiratory tract infections and pneumonia [27].

The elevated risk of gastrointestinal events indicates that patients with a history of gastrointestinal conditions should be closely monitored while receiving upadacitinib. Moreover, the higher risk of cancer associated with upadacitinib requires caution in patients with a history of malignancy or in those at an increased risk for cancer. Recent studies have observed an increased incidence of malignancies in patients treated with upadacitinib, raising concerns regarding its long-term safety [28].

The distinct safety profiles observed among the three JAK inhibitors suggest that different medications may be more suitable for different patient populations. For patients with cardiovascular risk factors, tofacitinib or upadacitinib might be preferable options over baricitinib due to the latter’s higher risk of cardiovascular events. However, considering the elevated risk of cancer associated with upadacitinib, tofacitinib may be the most appropriate choice in such scenarios. Tofacitinib, with its lower associated risk of cancer, may be a safer option for patients with a history of malignancy or those at a higher risk for cancer.

Conversely, for patients with gastrointestinal diseases, baricitinib, which showed a lower ROR for gastrointestinal-related events compared to tofacitinib and upadacitinib, may be preferable. Patients with pre-existing respiratory conditions might be better managed with tofacitinib or baricitinib because of the higher risk of respiratory adverse events associated with upadacitinib.

Our demographic analysis revealed that women represented the majority of reported cases across all three drugs, reflecting the higher prevalence of RA among females [29]. This gender disparity underscores the importance of considering sex-specific factors when managing and monitoring AEs among RA patients.

The high rates of hospitalization and serious outcomes associated with all three JAK inhibitors highlight the need for vigilant monitoring. Hospitalizations were notably higher for tofacitinib (27,399 reports), baricitinib (2257 reports), and upadacitinib (6275 reports). Disability outcomes and life-threatening events were also significant. These findings emphasize the importance of patient education regarding the potential risks and the need for prompt reporting of adverse events.

Our analysis directly compared the safety profiles of the three JAK inhibitors using real-world data from a large adverse events reporting system. Although prior research has examined the safety of individual JAK inhibitors, few studies have offered head-to-head comparisons. For instance, the Oral Rheumatoid Arthritis Trial (ORAL) Surveillance study compared comparing tofacitinib with tumor necrosis factor (TNF) inhibitors and found an increased risk of cardiovascular events and malignancies with tofacitinib. However, this study did not include baricitinib or upadacitinib [13]. Our findings align with the ORAL Surveillance study regarding the increased risk of serious AEs associated with JAK inhibitors but extend the comparison to include all three agents. In addition, a network meta-analysis by Schipper et al. evaluated the safety of various biological agents. They targeted synthetic DMARDs in RA and found differences in safety profiles among the agents, supporting the need for individualized treatment decisions [30].

Several limitations of this study should be acknowledged. This study is limited by the retrospective nature of the FAERS data, which introduces the potential for selection bias as patient populations receiving each JAK inhibitor may differ significantly. The FAERS database relies on spontaneous reporting, which may lead to under-reporting or reporting biases. Additionally, the lack of data on the duration of drug use, concomitant medications such as aspirin or statins, and patient comorbidities limits the ability to attribute adverse events to specific drugs. The data cannot establish causality, as they only signal potential associations. Confounding factors, such as patients’ comorbidities, concomitant medications, and disease severity, were not fully captured in the database. Despite these limitations, FAERS provides valuable post-marketing surveillance data that can highlight potential safety concerns and warrant further investigation.

Future research should prioritize prospective cohort studies and randomized controlled trials to confirm these findings and to establish causal relationships. Pharmacogenomic studies may also help to identify genetic factors that predispose patients to adverse events, enabling personalized medicine approaches. Additionally, long-term safety data are needed to fully understand the risks associated with prolonged use of JAK inhibitors.

## Conclusion

This study highlights the critical need for individualized risk assessment when prescribing JAK inhibitors for RA treatment. The distinct safety profiles of tofacitinib, baricitinib, and upadacitinib suggest that patient-specific factors should guide medication selection. Clinicians should balance the benefits of symptom control with the potential risks of adverse events and tailor treatment plans to optimize patient outcomes. Vigilant monitoring and patient education are essential to enhance the safe use of these medications in clinical practice.

## Data Availability

The datasets used and analyzed in this study are available from the corresponding author upon reasonable request.

## References

[1] Venetsanopoulou AI et al (2023) Epidemiology and risk factors for rheumatoid arthritis development. Mediterr J Rheumatol 34(4):404–413 38282942 10.31138/mjr.301223.eaf PMC10815538

[2] Almutairi KB et al (2021) The prevalence of rheumatoid arthritis: a systematic review of population-based studies. J Rheumatol 48(5):669–676 33060323 10.3899/jrheum.200367

[3] Romão VC, Fonseca JE (2021) Etiology and risk factors for rheumatoid arthritis: a state-of-the-art review. Front Med (Lausanne) 8:689698 34901047 10.3389/fmed.2021.689698 PMC8661097

[4] Kahlenberg JM, Fox DA (2011) Advances in the medical treatment of rheumatoid arthritis. Hand Clin 27(1):11–20 21176795 10.1016/j.hcl.2010.09.002 PMC3135413

[5] Rehman SU et al (2024) Advancing rheumatic disease treatment: a journey towards better lives. Open Health 5(1). 10.1515/ohe-2023-0040

[6] Rai V et al (2023) Futuristic novel therapeutic approaches in the treatment of rheumatoid arthritis. Cureus 15(11):e49738 38161868 10.7759/cureus.49738 PMC10757589

[7] Solitano V et al (2023) Advanced combination treatment with biologic agents and novel small molecule drugs for inflammatory bowel disease. Gastroenterol Hepatol (N Y) 19(5):251–263 37799456 PMC10548249

[8] Kubo S, Nakayamada S, Tanaka Y (2023) JAK inhibitors for rheumatoid arthritis. Expert Opin Investig Drugs 32(4):333–344 37014106 10.1080/13543784.2023.2199919

[9] Szekanecz Z et al (2024) Efficacy and safety of JAK inhibitors in rheumatoid arthritis: update for the practising clinician. Nat Rev Rheumatol 20(2):101–115 38216757 10.1038/s41584-023-01062-9

[10] Mansilla-Polo M, Morgado-Carrasco D (2024) Biologics versus JAK inhibitors. Part I: cancer risk. A narrative review. Dermatol Ther 14(6):1389–1442 10.1007/s13555-024-01166-4 PMC11169156 38763966

[11] Hoisnard L et al (2022) Adverse events associated with JAK inhibitors in 126,815 reports from the WHO pharmacovigilance database. Sci Rep 12(1):7140 35504889 10.1038/s41598-022-10777-w PMC9065106

[12] Mytheen S et al (2023) Investigating the risk of deep vein thrombosis with JAK inhibitors: a disproportionality analysis using FDA Adverse Event Reporting System Database (FAERS). Expert Opin Drug Saf 22(10):985–994 37294921 10.1080/14740338.2023.2223955

[13] Ytterberg SR et al (2022) Cardiovascular and cancer risk with tofacitinib in rheumatoid arthritis. N Engl J Med 386(4):316–326 35081280 10.1056/NEJMoa2109927

[14] Yang V et al (2023) Managing cardiovascular and cancer risk associated with JAK inhibitors. Drug Saf 46(11):1049–1071 37490213 10.1007/s40264-023-01333-0 PMC10632271

[15] Verden A et al (2018) Analysis of spontaneous postmarket case reports submitted to the FDA regarding thromboembolic adverse events and JAK inhibitors. Drug Saf 41(4):357–361 29196988 10.1007/s40264-017-0622-2

[16] Dong Z et al (2022) Thromboembolic events in Janus kinase inhibitors: a pharmacovigilance study from 2012 to 2021 based on the Food and Drug Administration’s Adverse Event Reporting System. Br J Clin Pharmacol 88(9):4180–4190 35466415 10.1111/bcp.15361

[17] Mogul A, Corsi K, McAuliffe L (2019) Baricitinib: the second FDA-approved JAK inhibitor for the treatment of rheumatoid arthritis. Ann Pharmacother 53(9):947–953 30907116 10.1177/1060028019839650

[18] Wu Y, Wei M, Zhang J (2023) A real-world pharmacovigilance analysis of FDA adverse event reporting system database for upadacitinib. Front Pharmacol 14. 10.3389/fphar.2023.1200254 10.3389/fphar.2023.1200254 PMC10469920 37663269

[19] Fang H et al (2014) Exploring the FDA adverse event reporting system to generate hypotheses for monitoring of disease characteristics. Clin Pharmacol Ther 95(5):496–498 24448476 10.1038/clpt.2014.17 PMC4194268

[20] FDA. Food and Drug Administration Adverse Event Reporting System (FAERS) quarterly data extract files. [cited 2024 Mar 29]; Available from: https://fis.fda.gov/extensions/FPD-QDE-FAERS/FPD-QDE-FAERS.html. Accessed 15 Oct 2024

[21] https://openvigil.pharmacology.uni-kiel.de/contingency-table-calculator.php. Accessed 15 Oct 2024

[22] Zou F, et al (2024) Adverse drug events associated with linezolid administration: a real-world pharmacovigilance study from 2004 to 2023 using the FAERS database. Front Pharmacol 15 10.3389/fphar.2024.1338902 PMC10904462 38434706

[23] Cohen SB et al (2020) Long-term safety of tofacitinib up to 9.5 years: a comprehensive integrated analysis of the rheumatoid arthritis clinical development programme. RMD Open 6(3):e001395 33127856 10.1136/rmdopen-2020-001395 PMC7722371

[24] Wollenhaupt J et al (2019) Safety and efficacy of tofacitinib for up to 9.5 years in the treatment of rheumatoid arthritis: final results of a global, open-label, long-term extension study. Arthritis Res Ther 21(1):89 30953540 10.1186/s13075-019-1866-2 PMC6451219

[25] Taylor PC et al (2022) Safety of baricitinib for the treatment of rheumatoid arthritis over a median of 4.6 and up to 9.3 years of treatment: final results from long-term extension study and integrated database. Ann Rheum Dis 81(3):335–343 34706874 10.1136/annrheumdis-2021-221276 PMC8862028

[26] U.S. Food and Drug Administration. FDA requires warnings about increased risk of serious heart-related events, c., blood clots, and death for JAK inhibitors. FDA Drug Safety Communication. September 1, 2021. Available at: https://www.fda.gov/drugs/drug-safety-and-availability/fda-requires-warnings-about-increased-risk-serious-heart-related-events-cancer-blood-clots-and-death

[27] Deodhar A et al (2022) Safety and efficacy of upadacitinib in patients with active ankylosing spondylitis and an inadequate response to nonsteroidal antiinflammatory drug therapy: one-year results of a double-blind, placebo-controlled study and open-label extension. Arthritis Rheumatol 74(1):70–80 34196498 10.1002/art.41911 PMC9299108

[28] Cohen SB et al (2021) Safety profile of upadacitinib in rheumatoid arthritis: integrated analysis from the SELECT phase III clinical programme. Ann Rheum Dis 80(3):304–311 33115760 10.1136/annrheumdis-2020-218510 PMC7892382

[29] Cross M et al (2014) The global burden of rheumatoid arthritis: estimates from the global burden of disease 2010 study. Ann Rheum Dis 73(7):1316–1322 24550173 10.1136/annrheumdis-2013-204627

[30] He Y et al (2013) Efficacy and safety of tofacitinib in the treatment of rheumatoid arthritis: a systematic review and meta-analysis. BMC Musculoskelet Disord 14:298 24139404 10.1186/1471-2474-14-298 PMC3819708

